# The relationship between symptomatic status and aneurysm wall enhancement characteristics of single unruptured intracranial aneurysm

**DOI:** 10.1007/s00117-024-01305-0

**Published:** 2024-04-30

**Authors:** Zi Chen, Wei Zhang, Fang-li Li, Wen-biao Lu

**Affiliations:** 1 Department of Radiology, Brain Hospital of Hunan Province, the School of Clinical Medicine, Hunan University of Chinese Medicine, No.427, Section 3, Furong Middle Road, Yuhua District, Changsha, Hunan Province China; 2 Department of Radiology, Hu’nan Prevention and Treatment Institute for Occupational Diseases, Changsha, China

**Keywords:** Intracranial aneurysm, Magnetic resonance imaging, Aneurysm wall enhancement, Intrakranielles Aneurysma, Magnetresonanztomographie, Aneurysmawandverstärkung

## Abstract

**Objective:**

We aimed to analyze the aneurysm wall enhancement (AWE) characteristics of a single unruptured intracranial aneurysm (UIA) and observe the relationship between the symptoms of a single UIA and the aneurysm wall.

**Methods:**

In our hospital, 85 patients diagnosed with a single UIA using computed tomography angiography (CTA) were retrospectively analyzed. The patients were divided into symptomatic and asymptomatic groups, including 46 asymptomatic and 39 symptomatic aneurysms. High-resolution magnetic resonance imaging of the vascular wall (HR-MR-VWI) was utilized to ascertain the presence, degree, and extent of AWE and thick-wall enhancement. In addition to AWE characteristics, morphological parameters of aneurysms, such as maximal size, shape, height, neck width, aspect ratio (AR), and size ratio (SR), were scanned using CTA. The differences in the parameters of a single UIA between the two groups were compared. An investigation explored the correlation between the symptom status of a single UIA and AWE.

**Results:**

We observed a correlation between symptom status and maximal size, height, and neck width for a single UIA, the presence or absence of AWE, and the levels and boundaries of AWE and thick-wall reinforcement. This study found that the AWE range was independently correlated with symptom status in the multivariate regression analysis.

**Conclusion:**

A larger AWE range was an independent risk factor for the onset of symptoms in a single UIA.

## Abstract (de)

**Ziel:**

Ziel der vorliegenden Arbeit war es, die Charakteristika der Aneurysmawandverstärkung (AWE) eines einzelnen nichtrupturierten intrakraniellen Aneurysmas (UIA) zu untersuchen und den Zusammenhang zwischen den Symptomen eines einzelnen UIA und der Aneurysmawand zu ermitteln.

**Methoden:**

In der Klinik der Autoren wurde bei 85 Patienten die Diagnose eines einzelnen UIA anhand einer Computertomographie-Angiographie (CTA) gestellt und eine retrospektive Analyse durchgeführt. Dabei wurden die Patienten in eine symptomatische und eine asymptomatische Gruppe unterteilt, mit 46 asymptomatischen und 39 symptomatischen Aneurysmen. Unter Verwendung der hochauflösenden Magnetresonanztomographie der Gefäßwand wurden das Vorliegen, der Grad und die Ausdehnung der AWE und der dickwandigen Erweiterung bestimmt. Zusätzlich zu den Merkmalen der AWE wurden morphologische Parameter der Aneurysmen, wie maximale Größe, Form, Höhe, Halsweite, Seitenverhältnis (AR) und Größenverhältnis (SR) mittels CTA untersucht. Zwischen den beiden Gruppen wurden die Unterschiede bei den Parametern eines einzelnen UIA verglichen. Dann wurde eine Untersuchung der Korrelation zwischen dem Symptomstatus bei einem einzelnen UIA und der AWE durchgeführt.

**Ergebnisse:**

Es wurde eine Korrelation zwischen dem Symptomstatus und der maximalen Größe, Höhe sowie Halsweite bei einem einzelnen UIA, dem Vorliegen oder Nichtvorhandensein einer AWE und den Ebenen und Grenzen von AWE und dickwandiger Verstärkung festgestellt. Ein Ergebnis der vorliegenden Studie war, dass die Spannbreite der AWE unabhängig mit dem Symptomstatus in der multivariaten Regressionsanalyse korreliert war.

**Schlussfolgerung:**

Eine größere AWE-Spannbreite war ein unabhängiger Risikofaktor für den Beginn von Symptomen bei einem einzelnen UIA.

## Introduction

Unruptured intracranial aneurysms (UIAs) affect approximately 3% of adults [1] and can be a major source of subarachnoid hemorrhage, a condition associated with a higher risk of death or life-altering disabilities [2–4]. Clinical symptoms primarily include headaches and cranial nerve palsy. Additionally, some patients may experience warning headaches, characterized by a severe headache that resolves within 72 h, 2 weeks before aneurysm rupture. A small amount of bleeding or deformed stretching of the aneurysm may cause this aura headache before the aneurysm ruptures [5]. Symptomatic and asymptomatic UIA comparisons revealed that the former had a significantly higher fracture risk than the latter, with an odds ratio of 4.4 (95% CI: 2.8–6.8; [6]). Symptomatic UIA has a higher risk of rupture and a relatively poor prognosis. However, the sensitivity and specificity of identifying symptomatic UIA based on clinical manifestations alone are insufficient, and more objective evidence is required.

In addition to symptoms, aneurysm wall enhancement (AWE) is more common in ruptured aneurysms than in UIAs [2] and has been observed in symptomatic or growing UIAs in some studies [7, 8]. This study used high-resolution magnetic resonance vascular wall imaging (HR-MR-VWI) to qualitatively analyze and rank AWE features of symptomatic UIAs. Through comprehensive multivariate analysis, this study explored the characteristics of the AWE that may be closely related to the condition of UIA in order to provide guidance for clinical treatment and contribute to improved outcomes.

## Materials and methods

### Patients

Comprehensive clinical data of 125 patients with UIAs diagnosed using computed tomography angiography (CTA) between January 2020 and June 2022 were collected at our hospital. Patients participating in this study met the following criteria: (1) patients with intracranial aneurysms diagnosed using CTA and (2) patients with comprehensive clinical and imaging data. The exclusion criteria were as follows: (1) patients with multiple unruptured aneurysms; (2) patients previously treated for an aneurysm; (3) patients diagnosed with subarachnoid hemorrhage caused by intracranial aneurysm rupture by CT, surgical puncture, or lumbar puncture; (4) patients with fusiform, traumatic, or saccular IA, and vascular malformations or low image resolution. Finally, 85 patients met the inclusion criteria. Clinical data collection included age, gender, hypertension, diabetes, hyperlipidemia, smoking and drinking habits, and symptoms.

Symptoms associated with aneurysms were defined as follows: (1) acute headache, sudden severe headache at onset, relieved after 72 h; (2) chronic headache, severe headache occurring for more than 4 h daily, 15 days per month, and 3 months consecutively; (3) cranial nerve symptoms associated with aneurysms, such as unilateral vision loss, diplopia, post-orbital pain, loss of pupillary light reflex, ptosis, eye extrusion paralysis, and trigeminal pain. All the aforementioned symptoms were determined to be aneurysm related by a combination of neurosurgeons, surgeons, and radiologists [9].

### Imaging protocol

#### Computed tomography angiography

The CTA was performed using a 128-slice CT scanner (Philips Ingenuity, the Netherlands) after injecting 60 mL iohexol containing 350 mg/mL of iodine into the antecubital vein at a rate of 5 mL/s. The scan range ranged from the level of the two cervical vertebrae to the top of the brain. The original image information was transmitted to a postprocessing workstation (Philips Nebula), where three-dimensional (3D) volume-rendered (VR) images and maximum intensity projection (MIP) images were obtained.

The following CTA parameters were used to scan for aneurysm localization: tube voltage = 120 kV; tube current = 250 mA; slice spacing = 0.5 mm; slice thickness = 1 mm; pitch = 0.891.

#### Magnetic resonance angiography

Magnetic resonance angiography (MRA) examinations were performed on all patients using a 3.0‑T scanner (Philips Ingria, the Netherlands) with eight-channel head coils. The examination included 3D time-of-flight (3D-TOF) MRA and contrast-enhanced pre- and post-optimized T1-weighted HR-MRI.

The 3D-TOF-MRA was performed using the following parameters for the localization of subsequent scans: TR/TE = 29/2 ms; field of view = 160 × 160 mm; intra-slice resolution = 0.5 × 0.8 mm; slice thickness = 1 mm. It took approximately 4 min and 46 s to convert these images into 3D VR and MIP images.

Pre- and post-contrast T1-weighted HR-MRI was performed using the following parameters: TR/TE = 1500/15 ms; field of view = 200 × 180 mm; acquired matrix = 384 × 224; and 0.5-mm isotropic resolution with a scan time of 6 min and 56 s per sequence. All patients were administered a single intravenous injection of 0.1 mmol/kg Gd-BOTPA (Magnevist, Bayer, Germany).

### Image analysis

A radiologist with 8 years of experience chose the best viewing angle to locate the aneurysm and measured the morphological parameters from the VR image of CTA. These parameters [10], including neck width, maximal size, height, average maximal size of the parent artery, aspect ratio, and size ratio, were clearly defined and described in the literature.

Image analysis of MRI was performed by a radiologist with 8 years of experience in CNS imaging diagnosis and who was blind to the clinical data. The AWE was defined as enhanced sites in post-contrast T1-weighted HR-MRI, such as the neck, body, apical, ascomycetes, or any strengthening of the entire tumor wall. The AWE type of each UIA was evaluated by comparing pre- and post-contrast T1-weighted HR-MRI. The enhancement grade was divided into three grades [11, 12]: 0 indicates no AWE, 1 indicates enhancement beyond the normal vessel wall, and 2 indicates enhancement equal to or greater than the pituitary infundibulum. The enhancement range was divided into four levels: level 0 (no AWE), level 1 (AWE less than 50% area), level 2 (AWE range 50–99% area), and level 3 (fully enhanced). Thick-walled enhancement was defined as enhanced aneurysm wall thickness ≥ 1 mm [13].

### Statistical analysis

The data were analyzed and visualized using SPSS 26.0. Enumeration data are described as number and percentage. Measurement data were tested for normality using the Shapiro–Wilk test, presenting a normal distribution pattern with a standard deviation of $\overline{\mathrm{x}}\pm \mathrm{s}$. The difference in the proportion between group A with symptoms and group B without symptoms was studied using the chi-square test. An independent-sample *t* test was used to compare the differences of normal indicators between the symptomatic and asymptomatic groups. A value of *p* < 0.05 was considered statistically significant when binary multivariate logistic regression analysis was used to analyze the influencing factors of symptoms.

## Results

Overall, 85 patients (females: 42, males: 43) met the inclusion criteria. This age group ranged from 45 to 70, averaging 58.69 ± 11.88. Among these patients, 39 were symptomatic and 46 were asymptomatic. Age, gender, smoking status, alcohol consumption, hypertension, diabetes, and hyperlipidemia did not differ significantly between the symptomatic and asymptomatic groups (*p* > 0.05). The baseline data between the two groups were balanced, indicating that the indicators in our study were comparable. Table 1 demonstrates the clinical features of the 85 patients.

**Table 1:** Clinical characteristics of patient with UIA^a^

Clinical data		Asymptomatic (*n* = 46)	Symptomatic (*n* = 39)	*p*
Age (Y)		59.10 ± 12.06	58.20 ± 11.80	0.730
Sex	Male	22 (47.8)	20 (51.3)	0.751
	Female	24 (52.2)	19 (48.7)	
Smoking	No	28 (63.9)	22 (56.5)	0.682
	Yes	18 (39.1)	17 (43.5)	
Alcohol	No	33 (71.7)	29 (74.3)	0.789
	Yes	13 (28.3)	10 (25.7)	
Hypertension	No	22 (47.8)	19 (48.7)	0.935
	Yes	24 (52.2)	20 (51.3)	
Diabetes	No	38 (82.6)	33 (84.6)	0.804
	Yes	8 (17.4)	6 (15.4)	
Hyperlipidemia	No	40 (87)	33 (84.6)	0.757
	Yes	6 (13)	6 (15.4)	

^a^$$\overline{\mathrm{x}}\pm \mathrm{s}$$, *n *(%)

The symptomatic group had a higher maximum size, height, and neck width than the asymptomatic group (*p* < 0.05, Table 2). The results showed that the proportion of UIAs with enhancement (79.5%) was higher than that of the asymptomatic control group (34.8%), with a statistically significant difference (Table 2). The enhancement characteristics of asymptomatic and symptomatic groups were also significantly different, including enhancement grade and range, as well as the difference in thickness enhancement rate (*p* < 0.001, Table 2).

**Table 2:** Differences in aneurysm-related indicators between control and symptomatic groups^a^

Variable		Asymptomatic (*n* = 46)	Symptomatic (*n* = 39)	*p*
Location	ICA	24 (52.2)	21 (53.8)	0.975
	MCA	9 (19.6)	8 (20.5)	
	ACA	3 (6.5)	3 (7.7)	
	PCA	10 (21.7)	7 (17.9)	
Maximal diameter (mm)		7.27 ± 3.53	10.01 ± 5.66	0.008
Height (mm)		7.62 ± 3.98	9.32 ± 3.19	0.035
Neck width (mm)		5.63 ± 3.44	7.66 ± 4.59	0.023
Lobes/ascomycetes	No	39 (84.8)	27 (69.2)	0.086
	Yes	7 (15.2)	12 (30.8)	
AR (aspect ratio)		1.33 ± 0.39	1.37 ± 0.41	0.646
SR (size ratio)		2.78 ± 1.17	2.85 ± 1.28	0.790
Enhancement	No	30 (65.2)	8 (20.5)	< 0.001
	Yes	16 (34.8)	31 (79.5)	
Enhancement grade	0	30 (65.2)	8 (20.5)	< 0.001
	1	8 (17.4)	9 (23.1)	
	2	8 (17.4)	22 (56.4)	
Enhancement range	0	30 (65.2)	5 (12.8)	< 0.001
	1	12 (26.1)	6 (15.4)	
	2	3 (6.5)	19 (48.7)	
	3	1 (2.2)	9 (23.1)	
Thick-walled enhancement	No	45 (97.8)	27 (69.2)	< 0.001
	Yes	1 (2.1)	12 (30.7)	

^a^$$\overline{\mathrm{x}}\pm \mathrm{s}$$, *n *(%)

*ACA* anterior cerebral artery, *AR* aspect ratio, *ICA* internal carotid artery, *MCA* middle cerebral artery, *PCA* posterior cerebral artery, *SR* size ratio

**Fig. 1:**
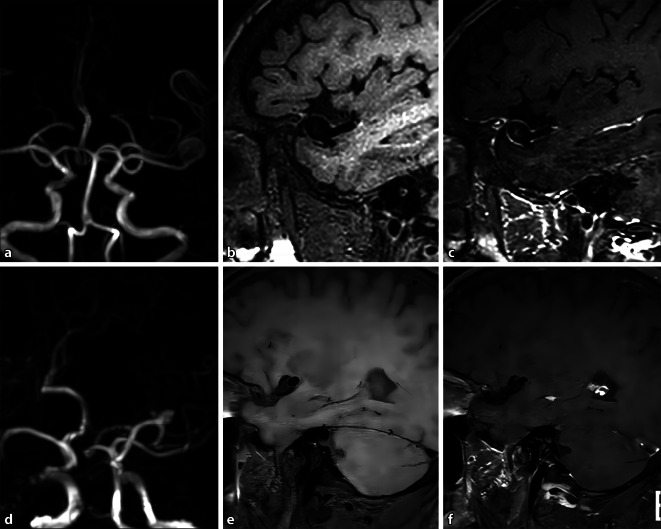
A 66-year-old female patient presented with a severe headache. **a** MRA displays the left middle cerebral artery UIA with a maximal size of 9.6 mm; **b** pre-contrast and **c** post-contrast sagittal MR-VWI presents an obvious (AWE degree, 2) and complete (AWE extent, 3) aneurysm wall enhancement. **d** MRA of a 68-year-old male patient exhibits an asymptomatic left middle cerebral artery UIA with a maximal size of 6.8 mm; **e** pre-contrast and **f** post-contrast sagittal MR-VWI demonstrates no enhancement of the aneurysm wall

**Fig. 2:**
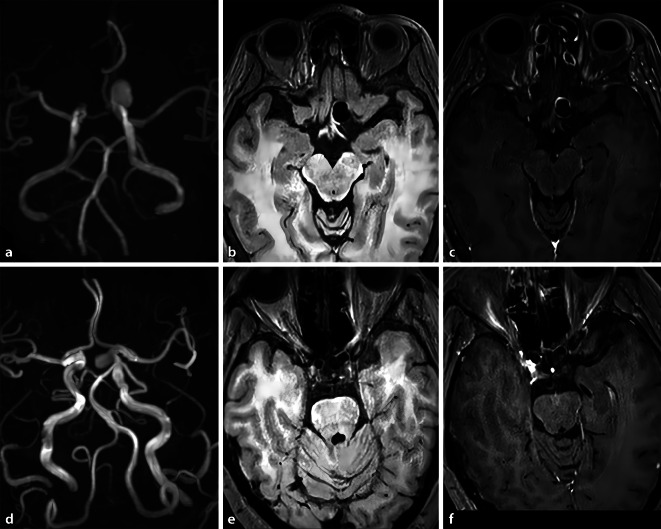
A 64-year-old female patient with decreased visual acuity in the left eye. **a** MRA exhibits the left internal carotid artery UIA with a maximal size of 11.7 mm; **b** pre-contrast and **c** post-contrast axial MR-VWI displays obvious (AWE extent, 2) and diffuse (AWE extent, 2) localized thick-walled enhancement. **d** MRA of a 41-year-old female patient displays an asymptomatic left internal carotid UIA with a maximal size of 7.2 mm; **e** pre and **f** post-enhanced axial MR-VWI depicts weak (AWE extent, 1) and limited (AWE extent, 1) aneurysm wall enhancement

The presence or absence of symptoms was used as the dependent variable (0 = none, 1 = yes), other variables were used as independent variables, and the influencing factors of the symptoms were analyzed. The results showed that as the grade of the enhancement range increased, the likelihood of symptoms occurring may increase (OR = 4.520; 95% CI: 1.037–8.074; Table 3). Figures 1 and 2 illustrate the HR-MR-VWI manifestations of symptomatic and asymptomatic UIAs at different locations.

**Table 3:** Symptomatic dichotomous multivariate logistic regression analysis

Variable	β	SE	Wald	*p*	OR	95% CI
Maximal size	−0.476	0.458	1.080	0.299	0.621	0.944 ～ 1.263
Height	0.550	0.364	2.280	0.131	1.733	0.922 ～ 1.297
Neck width	0.232	0.364	1.364	0.243	1.261	0.915 ～ 1.275
Enhancement	0.075	1.521	0.002	0.961	1.077	0.079 ～ 35.668
Enhancement grade	−0.681	0.895	0.579	0.447	0.506	0.217 ～ 5.660
Enhancement range	1.508	0.625	5.820	0.016	4.520	1.037 ～ 8.074
Thick-walled enhancement	1.785	1.279	1.947	0.163	5.959	1.257 ～ 74.848

*CI* confidence interval, *OR* odds ratio, *SE* standard error

## Discussion

This study analyzed the correlation between the symptom status of a single UIA and AWE. The results showed that the symptom status of a single UIA was related to the maximal size, AWE, enhancement degree, enhancement range, and thick-wall enhancement of UIA. Multivariate regression analysis demonstrated that only the range of AWE was independently related to symptom status. If the imaging features of diffuse and annular AWE on HR-MR-VWI can help identify symptomatic UIA, patients can receive timely and effective clinical interventions and treatments.

Recent research has reported that AWE in HR-MR-VVI plays a major role in infiltrating inflammatory cells, vascular constriction, thrombus formation, and weakening vascular elasticity [14, 15]. The inflammatory effect may cause endothelial cell and smooth muscle cell rupture injury, destroying the elastic layer in the tube wall, remodeling the tube wall, aggravating wall damage, and increasing the risk of rupture. The more pronounced the AWE, the less stable the aneurysm [16]. According to Edjlal et al. [13], the AWE characteristics of 263 IA patients showed that circumferential aneurysmal wall enhancement (CAWE) had high specificity (84.4%) and a high negative predictive value (94.3%) for identifying unstable IA. Omodaka et al. [17] indicated that the number of aneurysms developing CAWE was significantly higher than stable Ias and lower than ruptured Ias. Similarly, Edjlali et al. [18] reported that CAWE was detected more frequently in unstable Ias than in stable Ias (27/31,87% vs. 22/77, 28.5%; *p* < 0.0001), indicating that Ias with a larger range of enhancement have higher instability. In a previous small study (*n* = 25; [19]), AWE was more common in patients with symptomatic UIA. However, other factors correlated with these symptoms cannot be verified statistically. Symptoms aneurysms usually tend to be larger than asymptomatic aneurysms. Combining our research, we can interpret these variables with the analysis, indicating that the enhancement range is the most meaningful parameter when comparing enhanced MR images. Wang et al. [20] observed 89 Ias in 80 patients and discovered that symptomatic Ias had higher enhancement. Recent explorations by a research team led by Zhu [9] revealed a remarkable correlation between the AWE area and the symptoms of UIA patients. This study result is consistent with our findings, but some patients in these studies had multiple aneurysms. All participants included in this study had a single UIA to exclude the influence of factors such as changes in the pathological structure of ruptured and multiple aneurysms.

Currently, there are obvious differences in evaluating UIA enhancements. A previous study defined AWE with or without enhancement [7]. Several studies are graded based on the pituitary infundibulum [21], and another study reported circumferential aneurysmal wall enhancement (CAWE) and thick-walled CAWE [13]. However, AWE signatures may indicate varying degrees of inflammation or nutrient vessel density in the vessel wall, requiring a detailed and comprehensive analysis. This study conducted a comprehensive and detailed analysis of enhancement characteristics, such as AWE, enhancement degree, enhancement range, and thick-wall enhancement, and discovered that the enhancement range and thick-wall enhancement were the most significant factors associated with symptoms. The histological findings were consistent. Quan et al. [22] performed a comparative analysis of the histopathological data of aneurysm wall specimens and HR-MR-VWI characteristics of aneurysm wall specimens in 54 patients with UIA undergoing surgery; the results depicted focal wall enhancement of the aneurysm. Both the annular enhancement and aneurysm wall were linked to the inflammatory reaction of the aneurysm wall, but the inflammation of the annular enhancement was more prominent than that of the focal enhancement. A histological study by Matsushige [23] demonstrated that diffuse or annular AWE may be due to the presence of an intraluminal thrombus with a loose fibrous reticular structure and many neutrophils at the rupture site, whereas focal AWE is likely to be the result of contrast agent stagnation in the loose fibrous network of fresh intraluminal thrombus. The above studies indicate that the histopathological characteristics of AWE are related to factors such as inflammatory cell infiltration, pathological vasa vasorum hyperplasia, and intravascular thrombosis. When the aneurysm wall undergoes an inflammatory reaction and the formation of pathological feeder vessels, the normal endothelial barrier is destroyed and the contrast agent penetrates it, thus displaying the AWE. The larger the AWE range, the more severe and extensive the inflammatory impact on the arterial walls, which may threaten the stability of the aneurysm.

Currently, the clinical management of UIA mainly depends on its size and location. Most clinicians do not hesitate to choose a surgical intervention for large or symptomatic aneurysms. However, our study can offer objective evidence to help patients in better clinical management of borderline cases, such as those with a maximal size of approximately 5–6 mm or with only nonspecific symptoms, such as headaches. Zwarzanyet et al. [16] proposed that despite small intracranial aneurysms, it is important to analyze the risk factors for rupture, suggesting that we should be vigilant against smaller UIAs with abnormal enhancement. Consequently, we recommend active surgical intervention when diffuse or annular enhancement appears on HR-MR-VWI in borderline cases.

Recent studies [24] have demonstrated that a portion of AWE is correlated with slow blood flow, and the implementation of a slow-flow suppression module can effectively reduce these artifacts. In their experiments, researchers observed that only a small fraction of AWE was affected by applying slow-flow suppression pulses, suggesting that most AWE may still represent true enhancement of the vessel wall itself. However, it should be noted that AWE and low wall shear stress are linked to an increased risk of aneurysm growth and rupture. Therefore, caution must still be exercised when detecting AWE related to slow-flow artifacts.

### Limitations

Our study has several limitations. First, all sample data were collected from the same region, and the sample size was relatively small. Larger sample sizes and multicenter studies are necessary in further studies. Second, this study did not use histopathological techniques to investigate whether there was an association between AWE and arterial aneurysm wall inflammation due to the difficulty of sample collection. Third, several studies [24] have reported that slow-flow artifacts may mimic AWE and that the blood suppression module can help reduce flow artifacts. This module was not used in this study, and slow-flow artifacts may interfere with the judgment of AWE. In the future, HR-MR-VWI can be combined with a blood suppression module to improve the accuracy of AWE judgment and analysis. Finally, the lack of post-processing software precluded the analysis of aneurysm hemodynamics in this study. Further investigations are warranted to explore the interplay between hemodynamics, aneurysm wall enhancement, and symptoms for improved clinical management of unruptured aneurysms.

## Conclusion

A larger range of aneurysm wall enhancement range is an independent risk factor for symptomatic single unruptured intracranial aneurysm (UIA). This factor may be a marker of UIA instability and may provide appropriate clinical workup and improve patient outcomes.

## Data Availability

The data that support the findings of this study are available from the corresponding author upon reasonable request.

## Declarations

The study was performed in accordance with the ethical standards as laid down in the 1964 Declaration of Helsinki and its later amendments or comparable ethical standards. This retrospective study was approved by the ethics committee of the Brain Hospital of Hunan Province. Informed consent was obtained from all individual participants included in the study.

The supplement containing this article is not sponsored by industry.

## References

[1] Thompson BG, Brown RD Jr, Amin-Hanjani S et al (2015) Guidelines for the management of patients with unruptured intracranial aneurysms: a guideline for healthcare professionals from the American heart association/American stroke association. Stroke 46(8):2368–2400 26089327 10.1161/STR.0000000000000070

[2] Wang X, Zhu C, Leng Y, Degnan AJ, Lu J (2019) Intracranial aneurysm wall enhancement associated with aneurysm rupture: a systematic review and meta-analysis. Acad Radiol 26(5):664–673 29908979 10.1016/j.acra.2018.05.005

[3] Santarosa C, Cord B, Koo A et al (2020) Vessel wall magnetic resonance imaging in intracranial aneurysms: principles and emerging clinical applications. Interv Neuroradiol 26(2):135–146 31818175 10.1177/1591019919891297 PMC7507220

[4] Maupu C, Lebas H, Boulaftali Y (2022) Imaging modalities for Intracranial aneurysm: more than meets the eye. Front Cardiovasc Med 9:793072 35242823 10.3389/fcvm.2022.793072 PMC8885801

[5] Gilard V, Grangeon L, Guegan-Massardier E et al (2016) Headache changes prior to aneurysmal rupture: a symptom of unruptured aneurysm. Neurochirurgie 62(5):241–244 27527623 10.1016/j.neuchi.2016.03.004

[6] Wermer MJ, van der Schaaf IC, Algra A, Rinkel GJ (2007) Risk of rupture of unruptured intracranial aneurysms in relation to patient and aneurysm characteristics: an updated meta-analysis. Stroke 38(4):1404–1410 17332442 10.1161/01.STR.0000260955.51401.cd

[7] Vergouwen M, Backes D, van der Schaaf IC et al (2019) Gadolinium enhancement of the aneurysm wall in unruptured intracranial aneurysms is associated with an increased risk of aneurysm instability: a follow-up study. Ajnr Am J Neuroradiol 40(7):1112–1116 31221634 10.3174/ajnr.A6105 PMC7048551

[8] Matsushige T, Shimonaga K, Ishii D et al (2019) Vessel wall imaging of evolving unruptured Intracranial aneurysms. Stroke 50(7):1891–1894 31167619 10.1161/STROKEAHA.119.025245

[9] Zhu C, Wang X, Eisenmenger L et al (2020) Wall enhancement on black-blood MRI is independently associated with symptomatic status of unruptured intracranial saccular aneurysm. Eur Radiol 30(12):6413–6420 32666320 10.1007/s00330-020-07063-6

[10] Wang GX, Zhang D, Wang ZP, Yang LQ, Zhang L, Wen L (2016) Risk factors for the rupture of bifurcation intracranial aneurysms using CT angiography. Yonsei Med J 57(5):1178–1184 27401649 10.3349/ymj.2016.57.5.1178 PMC4960384

[11] Zhu C, Wang X, Degnan AJ et al (2018) Wall enhancement of intracranial unruptured aneurysm is associated with increased rupture risk and traditional risk factors. Eur Radiol 28(12):5019–5026 29872913 10.1007/s00330-018-5522-z

[12] Zhong W, Du Y, Guo Q et al (2020) The clinical and morphologic features related to aneurysm wall enhancement and enhancement pattern in patients with anterior circulation aneurysms. World Neurosurg 134:e649–e656 31689567 10.1016/j.wneu.2019.10.156

[13] Edjlali M, Guédon A, Ben Hassen W et al (2018) Circumferential thick enhancement at vessel wall MRI has high specificity for Intracranial aneurysm instability. Radiology 289(1):181–187 29969070 10.1148/radiol.2018172879

[14] Larsen N, von der Brelie C, Trick D et al (2018) Vessel wall enhancement in unruptured intracranial aneurysms: an indicator for higher risk of rupture? High-resolution MR imaging and correlated histologic findings. AJNR Am J Neuroradiol 39(9):1617–1621 30026386 10.3174/ajnr.A5731 PMC7655285

[15] Larsen N, Flüh C, Saalfeld S et al (2020) Multimodal validation of focal enhancement in intracranial aneurysms as a surrogate marker for aneurysm instability. Neuroradiology 62(12):1627–1635 32681192 10.1007/s00234-020-02498-6 PMC7666674

[16] Zwarzany Ł, Tyburski E, Poncyljusz W (2021) High-resolution vessel wall magnetic resonance imaging of small unruptured Intracranial aneurysms. J Clin Med 10(2) 10.3390/jcm10020225 PMC7827782 33435180

[17] Omodaka S, Endo H, Niizuma K et al (2018) Circumferential wall enhancement in evolving intracranial aneurysms on magnetic resonance vessel wall imaging. J Neurosurg: 1–7 10.3171/2018.5.JNS18322 30485237

[18] Edjlali M, Gentric JC, Régent-Rodriguez C et al (2014) Does aneurysmal wall enhancement on vessel wall MRI help to distinguish stable from unstable intracranial aneurysms. Stroke 45(12):3704–3706 25325912 10.1161/STROKEAHA.114.006626

[19] Fu Q, Guan S, Liu C, Wang K, Cheng J (2018) Clinical significance of circumferential aneurysmal wall enhancement in symptomatic patients with unruptured Intracranial aneurysms: a high-resolution MRI study. Clin Neuroradiol 28(4):509–514 28656370 10.1007/s00062-017-0598-4

[20] Wang GX, Gong MF, Zhang D et al (2019) Wall enhancement ratio determined by vessel wall MRI associated with symptomatic intracranial aneurysms. Eur J Radiol 112:88–92 30777225 10.1016/j.ejrad.2019.01.016

[21] Liu X, Zhang Z, Zhu C et al (2020) Wall enhancement of intracranial saccular and fusiform aneurysms may differ in intensity and extension: a pilot study using 7‑T high-resolution black-blood MRI. Eur Radiol 30(1):301–307 31218429 10.1007/s00330-019-06275-9

[22] Quan K, Song J, Yang Z et al (2019) Validation of wall enhancement as a new imaging biomarker of unruptured cerebral aneurysm. Stroke 50(6):1570–1573 31035900 10.1161/STROKEAHA.118.024195

[23] Matsushige T, Shimonaga K, Mizoue T et al (2019) Focal aneurysm wall enhancement on magnetic resonance imaging indicates Intraluminal thrombus and the rupture point. World Neurosurg 127:e578–e584 30928597 10.1016/j.wneu.2019.03.209

[24] Kalsoum E, Chabernaud Negrier A, Tuilier T et al (2018) Blood flow mimicking aneurysmal wall enhancement: a diagnostic pitfall of vessel wall MRI using the postcontrast 3D turbo spin-echo MR imaging sequence. AJNR Am J Neuroradiol 39(6):1065–1067 29599170 10.3174/ajnr.A5616 PMC7410621

